# Draft Genome Sequences of Six *Mycobacterium immunogenum* Strains Obtained from a Chloraminated Drinking Water Distribution System Simulator

**DOI:** 10.1128/genomeA.01538-15

**Published:** 2016-01-07

**Authors:** Vicente Gomez-Alvarez, Randy P. Revetta

**Affiliations:** U.S. Environmental Protection Agency, Office of Research and Development, Cincinnati, Ohio, USA

## Abstract

We report here the draft genome sequences of six *Mycobacterium immunogenum* strains isolated from a chloraminated drinking water distribution system simulator subjected to changes in operational parameters. *M. immunogenum*, a rapidly growing mycobacterium previously reported to be the cause of hypersensitivity pneumonitis from contaminated metalworking fluid aerosols, is becoming a public health concern.

## GENOME ANNOUNCEMENT

Many water utilities are considering changing from free chlorine to monochloramine to ensure regulatory compliance of disinfectant by-products (DBPs) in drinking water distribution systems (DWDS) (1). While all disinfection strategies aim to mitigate the presence of pathogens, they do not completely eradicate the growth of microorganisms in DWDS. Indeed, a diverse and distinct microbial community has been shown to inhabit DWDS (2), with a higher distribution of nontuberculous mycobacteria (NTM) in chloraminated systems (3). NTM are opportunistic pathogens with the ability to form biofilms in DWDS (4). In spite of the public health relevance of NTM, e.g., *Mycobacterium chelonae-M. abscessus* complex (5, 6), very little information is available about their occurrence in DWDS.

*Mycobacterium immunogenum* is a rapidly growing NTM and member of the *M. chelonae-M. abscessus* complex (7) that has long been implicated in hypersensitivity pneumonitis in metalworking workers (8). In addition, *M. immunogenum* has more recently been identified in a broad range of clinical cases (9), such as infectious keratitis (10), and recently from a brain abscess (11).

Strains from this study were isolated from bulk water and biofilms (polyvinyl chloride [PVC] or copper [Cu] surface) obtained from a chloraminated DWDS simulator (4). Samples were collected from three distinct operational schemes (Table 1). Colonies were obtained from R2A plates after 7 days at 27°C. DNA was extracted using the UltraClean DNA microbial isolation kit, according to the manufacturer’s instructions (MoBio Laboratories, Solana Beach, CA). Paired-end 125 bp libraries were prepared using the Nextera XT DNA library kit, followed by rapid mode sequencing on the HiSeq 2500 platform (Illumina, Inc., San Diego, CA).

**TABLE 1:** Summary statistics of whole-genome assemblies

Strain	Source (surface)	Operational scheme^a^	Fold coverage (×)	No. of contigs	Contig *N*_50_	Assembly size (bp)	G + C content (%)	Accession no.
H008	Biofilm (Cu)	Standard I	83	46	254,352	5,719,131	64.03	LJFO00000000
H068	Biofilm (Cu)	Failure	85	45	294,203	5,649,917	64.14	LJFQ00000000
H088	Biofilm (PVC)	Standard II	47	91	137,277	5,988,570	64.12	LJFT00000000
H097	Biofilm (Cu)	Standard II	41	77	169,953	5,810,596	64.01	LJFU00000000
HXV	Bulk water	Failure	104	46	254,354	5,679,282	64.14	LJFX00000000
HXXI	Bulk water	Standard II	51	89	147,477	5,975,867	64.12	LJFY00000000

a Standard I, stable chloramine residual; Failure, complete nitrification and minimal chloramine residual; Standard II, stable chloramine residual after a “chlorine-burn.”

Prior to assembly, the libraries were (i) cleaned of contaminants (adapters, *phiX*, artifacts, and human), (ii) error corrected via Tadpole, (iii) normalized to ≤100×, (iv) removed of low-coverage (<6×) reads, and (v) filtered to a minimum length read of 125 nucleotides (nt). The reads were processed using the software package BBMap version 35.34 (http://sourceforge.net/projects/bbmap). The processed reads were *de novo* assembled using the software SPAdes version 3.5.0 (12). The final assembly attributes are listed in Table 1. The average nucleotide identity (ANI), a similarity index between two genomes (13), revealed a genome similarity between the isolates of 99.901% to 99.998%. The proposed cutoff for species is 95% to 96% (14). The isolates share an average of 99.922% ANI with *M. immunogenum* SMUC14 (11) and 85.993% and 83.193% ANI with *M. abscessus* ATCC 19977 and *M. chelonae* ATCC 35752, respectively. The ANI calculations were performed using the online calculator available from EzGenome (http://www.ezbiocloud.net/ezgenome/ani).

Genome assemblies were annotated with Prokka version 1.10 (15), which is available as an application in Illumina BaseSpace Labs. The genome sequence of strain H008 contains 5,813 genes, 5,742 coding sequences (CDSs), 3 rRNAs, and 68 tRNAs; strain H068 contains 5,685 genes, 5,614 CDSs, 6 rRNAs, and 65 tRNAs; strain H088 contains 6,112 genes, 6,010 CDSs, 3 rRNAs, and 99 tRNAs; strain H097 contains 5,954 genes, 5,883 CDSs, 3 rRNAs, and 68 tRNAs; strain HXV contains 5,715 genes, 5,647 CDSs, 3 rRNAs, and 65 tRNAs; and strain HXXI contains 6,096 genes, 5,994 CDSs, 3 rRNAs, and 99 tRNAs.

### Nucleotide sequence accession numbers.

This whole-genome shotgun project has been deposited in DDBJ/ENA/GenBank under the accession numbers listed in Table 1. The versions described in this paper are the first versions.
